# Rapid and high-efficiency generation of mature functional hepatocyte-like cells from adipose-derived stem cells by a three-step protocol

**DOI:** 10.1186/s13287-015-0181-3

**Published:** 2015-10-05

**Authors:** Fen Xu, Junli Liu, Jie Deng, Xiaolei Chen, Yuan Wang, Pengchao Xu, Lin Cheng, Yanli Fu, Fuyi Cheng, Yunqi Yao, Yujing Zhang, Meijuan Huang, Dechao Yu, Yuquan Wei, Hongxin Deng

**Affiliations:** State Key Laboratory of Biotherapy and Cancer Center, West China Hospital, Sichuan University, and Collaborative Innovation Center for Biotherapy, No.17, section3, Renmin South Road, Chengdu, 610041 P.R. China; Department of Thoracic Oncology, Cancer Center, West China Hospital, Sichuan University, No. 37 Guo Xue Xiang, Chengdu, Sichuan 610041 P.R. China

## Abstract

The generation of functional hepatocytes is a major challenge for regenerative medicine and drug discovery. Here we show a method that facilitates generation of induced functional hepatocytes (iHeps) from adipose-derived stem cells (ADSCs) within 9 days. iHeps express hepatocytic gene programs and display functions characteristic of mature hepatocytes, including cytochrome P450 enzyme activity. Upon transplantation into mice with carbon tetrachloride (CCl_4_)-induced acute fulminant liver failure, iHeps restore the liver function and prolong survival. The work could contribute to the development of alternative strategies to obtain nonhepatic cell-derived mature hepatocytes with potential for biomedical and pharmaceutical applications.

**Electronic supplementary material:**

The online version of this article (doi:10.1186/s13287-015-0181-3) contains supplementary material, which is available to authorized users.

## Introduction

Functional hepatocytes are in high demand in the field of regenerative medicine and drug development. They show great potential for repairing or replacing diseased and damaged tissues and can be valuable tools for pharmaceutical applications. However, the practical application of primary hepatocytes has been frustrated by their tendency to rapidly dedifferentiate and lose most hepatic functions after growth in a tissue culture environment [1–4]. To solve this dilemma, novel strategies for generating sufficient hepatocytes are in high demand. In the last few years, extrahepatic cell populations with potential to generate functional hepatocytes have been discovered [5–8]. Currently, attention is being given to mesenchymal stem cells (MSCs) [9, 10], which can be obtained from different sources such as bone marrow [11], amniotic fluid [12], umbilical cord blood [13], scalp tissue [14], placenta [15], or adipose tissue [16] of the body. These cells show the ability of multipotentiality and semi-infinite proliferation. In particular, adipose-derived stem cells (ADSCs) are recognized as one of the most promising MSCs identified thus far, since adipose tissue is ubiquitous and easily obtained in large quantities with little donor site morbidity or discomfort [17–20]. Furthermore, recent research has revealed that the use of ADSCs in regenerative medicine is not limited to mesodermal tissue but extended to both ectodermal and endodermal tissues and organs, although ADSCs originate from mesodermal lineages [21–25].

In the present study, we describe the generation of induced functional hepatocytes (iHeps) from rat ADSCs in a rapid and high-efficiency manner. iHeps express hepatocytic gene programs and possess the functional properties of mature hepatocytes, including albumin (ALB) secretion, urea synthesis, and cytochrome P (CYP) 450 enzyme activity. Notably, we demonstrate the therapeutic effects of iHeps on carbon tetrachloride (CCl_4_)-induced acute fulminant liver failure. These results indicate that iHeps could be applied in cellular therapies, disease modeling, and drug discovery.

## Materials and methods

### Isolation, culture, and identification of rat ADSCs

Isolation of ADSCs was performed as described previously [26] with some modifications. The inguinal fat pad was collected under sterile conditions from 5-week-old female Lewis rats (Vital River Laboratory, Chengdu, Sichuan, P.R. China) and washed with Hank’s balanced salt solution (HBSS; Gibco, Chengdu, Sichuan, P.R. China). The washing step was usually repeated three times. Adipose tissue sample was minced into small pieces and digested in 0.1 % collagenase type I (3 ml for each 1 g tissue; Gibco) at 37 °C for 1 hour with a rotation speed of 120 rpm. After digestion, an equal volume of low-glucose Dulbecco’s modified Eagle’s medium (DMEM-LG; Gibco) containing 10 % fetal bovine serum (FBS; Gibco) was added. The cell suspension was filtered through a 100 μm filter (BD Falcon, Chengdu, Sichuan, P.R. China) for the removal of the solid aggregates. The sample was subsequently centrifuged at 1500 rpm for 10 minutes at 4 °C and completed the separation of the stromal cells from the adipocytes. The centrifugation step was repeated. The cells were resuspended in complete medium (DMEM-LG with 10 % FBS, 100 U/ml penicillin, and 100 μg/ml streptomycin) in a 75 cm^2^ culture dish and were maintained at 37 °C in saturated humidity with 5 % carbon dioxide. After 1 day, nonadherent cells were removed by two or three washes with HBBS and medium changes were performed every 2 days thereafter. Cell morphology was monitored under an inverted microscope. Passage 3 cells were used for flow cytometry analysis and differentiation assays.

For flow cytometry analysis, 5 × 10^5^ ADSCs (in 100 μl phosphate-buffered saline (PBS)) were incubated with different fluorescently labeled monoclonal antibodies (anti-rat CD45-PECy5, anti-rat CD31-PE, anti-rat CD29-FITC, anti-rat CD44H-FITC, and anti-rat CD90-FITC; Biolegend, Chengdu, Sichuan, P.R. China) and incubated in the dark at 2–8 °C for 30 minutes. After washing twice with PBS, cells were resuspended in 300 μl PBS and analyzed by the Calibur flow cytometer (BD Biosciences, Chengdu, Sichuan, P.R. China).

For adipocytic differentiation, ADSCs (5 × 10^3^ cells/cm^2^) were seeded in six-well plates. When ~100 % confluent, cells were maintained in adipocyte genesis medium (PT-3004; Lonza, Chengdu, Sichuan, P.R. China) for 2 weeks. Cells were then stained with Oil red O.

For osteogenic differentiation, ADSCs (5 × 10^3^ cells/cm^2^) were seeded in six-well plates. When ~100 % confluent, cells were treated with inducing medium (PT-3002; Lonza) for 3 weeks. Cells were then fixed with 4 % formaldehyde for 30 minutes and stained with Alizarin Red.

For chondrogenic differentiation, ADSCs were seeded at a density of 10^6^ per well in an ultralow attachment six-well plate, and cultured with inducing medium (PT-3003; Lonza) for 3 weeks. Cells were then identified by Alcian blue staining.

### In vitro differentiation of ADSCs into iHeps

At passages 3–7, the cells were seeded on collagen type I-coated culture dishes at a concentration of 2.0 × 10^4^ cells/cm^2^. When the cells reached 100 % confluence, hepatic induction was carried out over a period of 9 days (Table 1). First, the cells were treated for 1 day of endodermal induction with RPMI-1640 (Gibco) supplemented with 100 nM IDE1 (Tocris, Chengdu, Sichuan, P.R. China) and 3 μΜ CHIR99021 (Selleckchem, Chengdu, Sichuan, P.R. China). During the next step, the culture medium was replaced with hepatogenic induction medium containing 100 nM IDE1, 20 ng/ml fibroblast growth factor 4 (FGF4; PeproTech, Chengdu, Sichuan, P.R. China), and 150 ng/ml hepatocyte growth factor (HGF; PeproTech). Finally, during the maturation step, the cells were cultured in Williams’ E (Gibco) supplemented with 50 ng/ml HGF, 20 ng/ml epidermal growth factor (EGF; PeproTech), 30 ng/ml oncostatin M (OsM; PeproTech), 2 × 10^−5^ mol/l dexamethasone (Dex; Sigma, Chengdu, Sichuan, P.R. China) and 1 × insulin–transferrin–selenium (ITS; Sigma).

**Table 1:** Hepatic lineage differentiation condition

	Induction period	Medium contents
Endodermal induction	Days 0–1	RPMI-1640, IDE1 (100 nM), CHIR99021 (3 μM)
Hepatogenic induction	Days 2–4	RPMI-1640, IDE1 (100 nM), FGF4 (20 ng/ml), HGF (150 ng/ml)
Maturation step	Days 5–9	Williams’ E, HGF (50 ng/ml), EGF (20 ng/ml), OsM (30 ng/ml), Dex (2 × 10^−5^ M), ITS (1×)

*DEX* dexamethasone, *EGF* epidermal growth factor, *FGF4* fibroblast growth factor 4, *HGF* hepatocyte growth factor, *ITS* insulin–transferrin–selenium, *OsM* Oncostatin M

### Flow cytometry analysis

For intracellular staining of ALB and alpha-1-antitrypsin (AAT), 5 × 10^5^ iHeps were harvested and fixed with 4 % paraformaldehyde for 30 minutes, and then permeabilized in staining buffer (BD Biosciences) for 10 minutes. Cells were then incubated with primary antibody—sheep anti-ALB (Bethyl, Chengdu, Sichuan, P.R. China) and rabbit anti-AAT (American Research Products, Chengdu, Sichuan, P.R. China)—for 30 minutes in staining buffer, followed by secondary antibody—dylight 488 conjugated donkey anti-rabbit IgG (Bethyl) and dylight 594 conjugated donkey anti-sheep IgG (Bethyl)—incubation for 30 minutes. Cells were analyzed by the Calibur flow cytometer (BD Biosciences).

For detecting of asialoglycoprotein receptor (ASGPR), 5 × 10^5^ iHeps (in 100 μl PBS) were incubated with monoclonal antibodies (anti-ASGPR1-PE; Santa Cruz, Chengdu, Sichuan, P.R. China) and incubated in the dark at 2–8 °C for 30 minutes. After washing twice with PBS, cells were resuspended in 300 μl PBS and analyzed by the Calibur flow cytometer (BD Biosciences).

### Immunofluorescence

For intracellular staining of ALB and AAT, the cells were fixed with 4 % paraformaldehyde for 15 minutes at room temperature and then incubated with PBS containing 0.2 % Triton X-100 (Sigma) for 15 minutes. Cells were then washed three times with PBS. After being blocked by 3 % bovine serum albumin (BSA) in PBS for 60 minutes at room temperature, cells were incubated with primary antibodies at 4 °C overnight, washed three times with PBS, and then incubated with appropriate fluorescence-conjugated secondary antibody for 60 minutes at 37 °C in the dark. Nuclei were stained with 4',6-diamidino-2-phenylindole (DAPI; Sigma). Primary and secondary antibodies were diluted in PBS containing 3 % BSA. Antibodies used for immunofluorescence are as follows: sheep anti-ALB (1:500; Bethyl), rabbit anti-AAT (1:1; Abcam, Chengdu, Sichuan, P.R. China), rabbit anti-hepatocyte nuclear factor alpha-4 (anti-HNF4α, 1:50; Santa Cruz), rabbit anti-Ki67 (1:100; Santa Cruz), dylight 488 conjugated donkey anti-rabbit IgG (1:200; Bethyl), dylight 594 conjugated donkey anti-sheep IgG (1:200; Bethyl), and Alexa Fluor 594 conjugated goat anti-rabbit IgG (1:100; ZSBG-BIO, Chengdu, Sichuan, P.R. China).

### Real-time quantitative PCR

Total RNA was isolated from ADSCs, iHeps, and primary rat hepatocytes (rHeps) using the Trizol Reagent (Sigma). Additional file 1 shows the sequences of both forward and reverse primers in more detail. In parallel, we analyzed the mRNA concentration of the rat housekeeping GAPDH as an internal control for normalization. The real-time monitoring of the PCR reaction, the precise quantification of the products in the exponential phase of the amplification, and the melting curve analysis were performed with the Bio-Rad CFX Manager software (Chengdu, Sichuan, P.R. China), as recommended by the manufacturer.

### Gene expression profile analysis

Total RNA was extracted and reverse transcript into cDNA. Samples were then hybridized to the Affymetrix Rat Gene 1.0ST (Chengdu, Sichuan, P.R. China) in accordance with the manufacturer’s instruction. Data were normalized by Partek Genomics Suite 6.6 (Affymetrix, Beijing, P.R. China) in default analysis settings. Normalized data were processed to the Kyoto Encyclopedia of Genes and Genomes (KEGG, Chengdu, Sichuan, China) for further pathway function analysis.

### Periodic acid–Schiff staining, acetylated low-density lipoprotein, and indocyanine green uptake assays, ALB ELISA, and urea synthesis

iHeps were stained by Periodic acid–Schiff (PAS; Sigma) following the manufacturer’s instructions. For the acetylated low-density lipoprotein (ac-LDL) uptake assay, iHeps were incubated with 10 mg/ml DiI-labeled ac-LDL (Life Technologies, Chengdu, Sichuan, China) at 37 °C for 1 hour and later washed three times with PBS. For the indocyanine green (ICG; Sigma) uptake assay, media of iHeps were changed with 1 mg/ml ICG and incubated at 37 °C for 1 hour, followed by washing three times with PBS. To determine ALB secretion in iHeps, culture supernatants were collected daily and determined by the rat ALB ELISA kit (Bethyl) according to the manufacturer’s instructions. Urea content was measured with diacetylmonoxime with a commercially available kit (StanBio Laboratory, Chengdu, Sichuan, China).

### iHep transplantation to CCl_4_-induced acute fulminant liver failure mice

NPG mice (6 weeks old, male; Beijing Vitalstar Biotechnology Co., Ltd, Chengdu, Sichuan, China) were injected with CCl_4_ (Sigma) at a dose of 0.5 ml/kg body weight through intraperitoneal injection. Eight hours after CCl_4_ treatment, ADSCs, iHeps, and rHeps (2 × 10^6^ cells/animal) were injected into the spleens of the mice. Meanwhile, a sham operation group was used as negative control. Blood and liver samples were collected after the surviving animals were sacrificed. Blood samples were used for blood biochemical analysis. Livers of recipient mice were embedded in Tissue Freezing Medium (Leica, Chengdu, Sichuan, China) and then frozen in liquid nitrogen. Cryostat sections (8 μm) were stained. All animal experiments were in conformity with NIH guidelines (NIH Pub. No. 85-23, revised 1996) and were approved by the Animal Care and Use Committee of Sichuan University, Chengdu, P.R. China.

### In vivo tumor transplantation assay

Five BALB/c nude mice (6 weeks old, female; HFK Bioscience, Beijing, China) were subcutaneously injected with HepG2 or iHeps (1 × 10^6^) on both sides of the back. These mice were sacrificed 8 weeks after transplantation. All animal experiments were in conformity with NIH guidelines (NIH Pub. No. 85-23, revised 1996) and were approved by the Animal Care and Use Committee of Sichuan University.

## Results and discussion

### Characterization of ADSCs

**Fig. 1:**
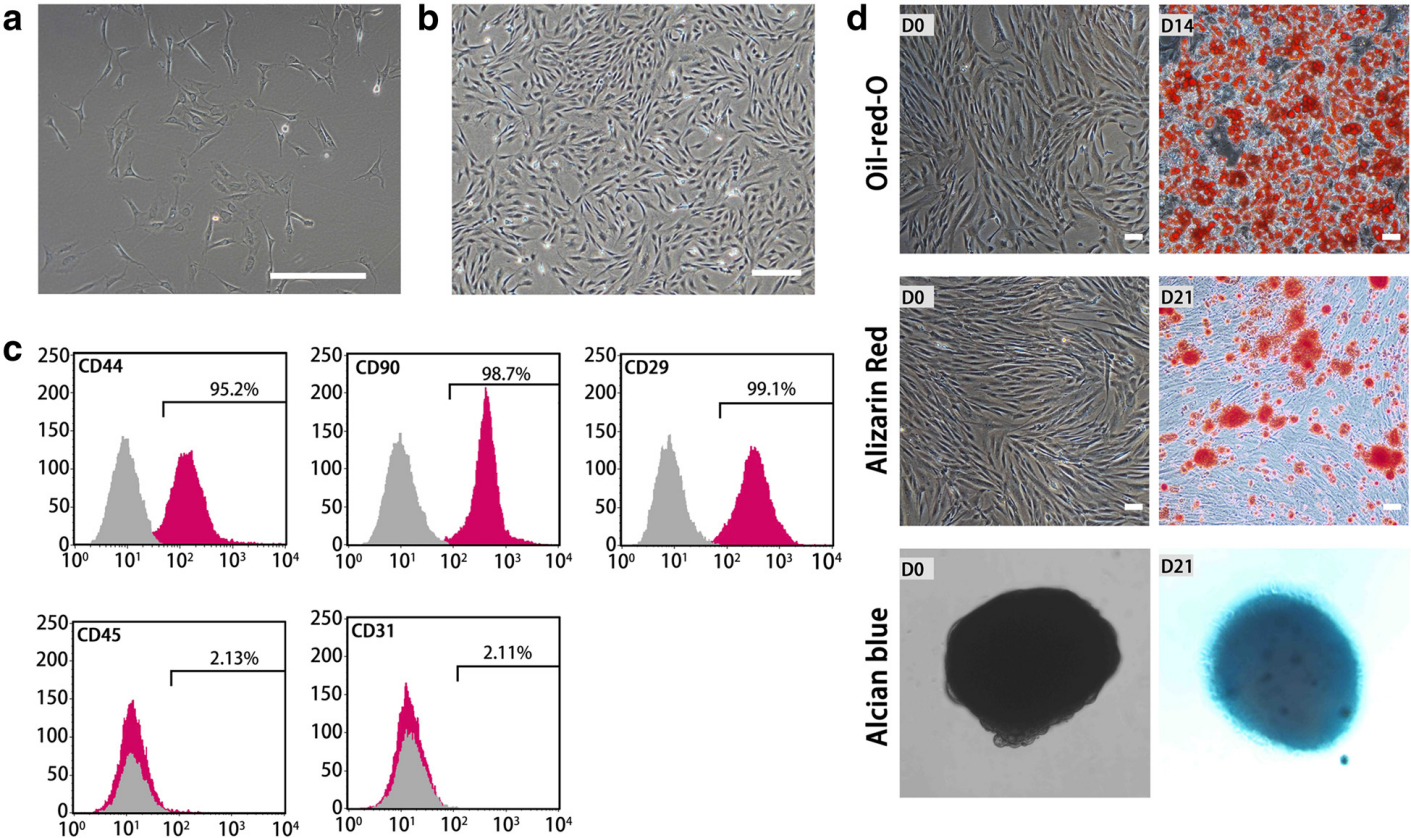
Characterization of rat ADSCs. **a** Primary ADSCs. **b** ADSCs in passage 3 with 90 % confluence. The cells showed homogeneous fibroblastic morphology. **c** Expression of cell surface markers on ADSCs. ADSCs were positive for mesenchymal stem markers (CD44, CD90, and CD29) and negative for hematopoietic marker (CD45) or endothelial marker (CD31). **d** Multiple differentiation potential of ADSCs. ADSCs could differentiate into adipocytes, osteocytes, or chondrocytes. Scale bar: 250 μm (**a**, **b**,) 100 μm (**d**)

The cultures were observed using an inverted light microscope. Attachment of spindle-shaped cells to the culture dish was observed after 1 day of culture (Fig. 1a). Primary cultures reached 80–90 % confluence in approximately 4–5 days. During passaging, the cell growth tended to accelerate and the morphology of cells changed gradually. After three passages, the cultures showed homogeneous fibroblastic morphology (Fig. 1b). Flow cytometry analysis showed that ADSCs possessed the surface marker profiles (CD29, CD44, and CD90) typical for MSCs, and were negative for hematopoietic marker (CD45) or endothelial marker (CD31) indicating the absence of hematopoietic and endothelial cells (Fig. 1c). When cultured in appropriate induction medium, ADSCs differentiated into adipocytes, which were verified by Oil red O staining (Fig. 1d). They were also able to differentiate into osteocytes, which were confirmed by Alizarin Red staining (Fig. 1d). Furthermore, they could differentiate into chondrocytes, which were identified by Alcian blue binding assay (Fig. 1d).

### Screening for induction protocols to generate iHeps from ADSCs

**Fig. 2:**
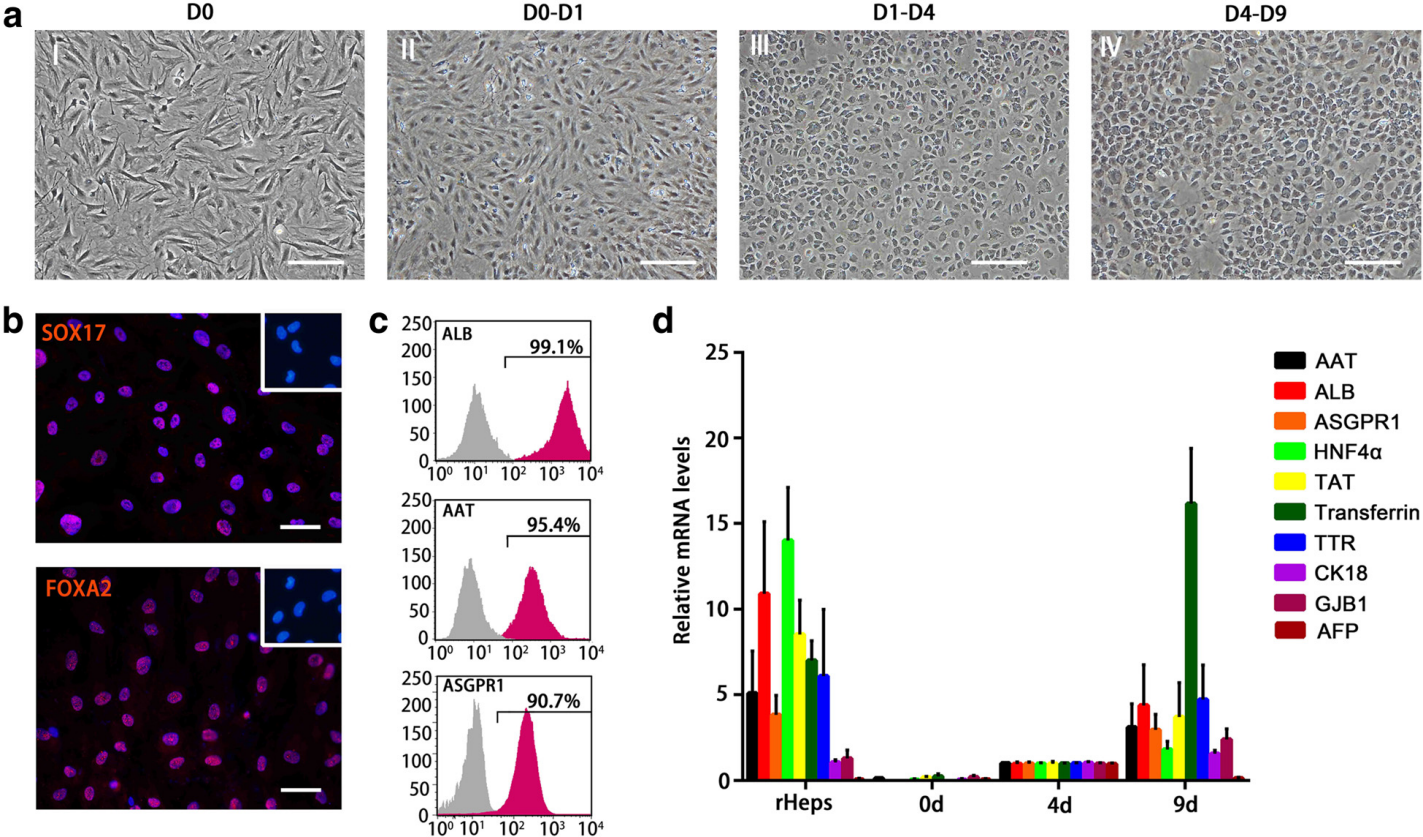
Screening for induction protocol to generate iHeps from ADSCs. **a** Sequential morphological changes from ADSCs to iHeps. *I* Cell morphology before hepatic lineage induction. *II* Change of cell morphology with a short spindle shape after culture in endodermal induction medium. *III* At day 4, the cell morphology had become polygonal in shape. *IV* Morphology of the mature iHeps. **b** Immunostaining revealed the vast majority of induced cells were positive for the DE markers Sox17 and FoxA2 at day 1 (*upper right*, ADSCs). **c** Differentiation efficiency measured by flow cytometry analysis marked by ALB, AAT, and ASGPR1. *n* = 3. **d** Gradual changes of hepatocytic gene expression in iHeps during hepatic lineage induction. The expression levels of the indicated genes were analyzed by quantitative PCR. Data are normalized to iHeps at day 4. *AAT* alpha-1-antitrypsin, *ALB* albumin, *ASGPR* asialoglycoprotein receptor, *CK18* cytokeratin 18, *d* day, *GJB1* gap junction protein beta-1, *HNF4α* hepatocyte nuclear factor alpha-4, *rHep* primary rat hepatocyte, *TAT* tyrosine transaminase, *TTR* transthyretin, *AFP* alpha fetal protein. Scale bar: 100 μm (**a**,) 50 μm (**b**). Data presented as mean ± standard deviation

For rapidly and highly efficiently generating mature hepatocytes from ADSCs, we screened a series of hepatogenic induction strategies and finally confirmed a three-step protocol which was described in Materials and methods. In this protocol, ADSCs were allowed to reach approximately 100 % confluence on collagen type I-coated dishes, and were then treated with endodermal induction medium on day 0 (Fig. 2a, I) in the presence of IDE1 and CHIR99021. After 1 day, the cell morphology turned from a long spindle shape into a short spindle shape (Fig. 2a, II). Immunofluorescent staining revealed that most of the cells were positive for the definitive endoderm (DE) markers Sox17 and FoxA2 (Fig. 2b), indicating that the ADSCs efficiently differentiated into DE during the endodermal induction step. Following the endodermal induction step, cells were treated with the hepatogenic induction medium for 3 days, which changed the cell morphology from a spindle shape to a polygonal shape (Fig. 2a, III). Finally, the medium was replaced with maturation medium which resulted in the cell morphology changing into a typical cuboidal shape of primary hepatocytes that had tight cell–cell contact (Fig. 2a, IV). Flow cytometry analysis of these hepatocyte-like cells (that is, iHeps) confirmed that nearly 100 % of iHeps were ALB-positive, ~95 % were AAT-positive, and ~91 % were ASGPR1-positive, indicating that iHeps possess these typical markers of mature hepatocytes (Fig. 2c). Furthermore, the expression of genes specific for mature hepatocytes (for example, ALB, ASGPR1, and Transferrin) increased gradually during iHep induction, while the expression of alpha fetal protein (AFP) for immature hepatocytes significantly upgraded in the hepatogenic induction stage and descended to the level of rHeps in the maturation step, suggesting that hepatic lineage differentiation is a progressively coordinated process (Fig. 2d). These results indicate that we successfully obtain ADSC-derived mature hepatocytes using a new procedure.

### iHeps possess the typical characteristics of mature hepatocytes

**Fig. 3:**
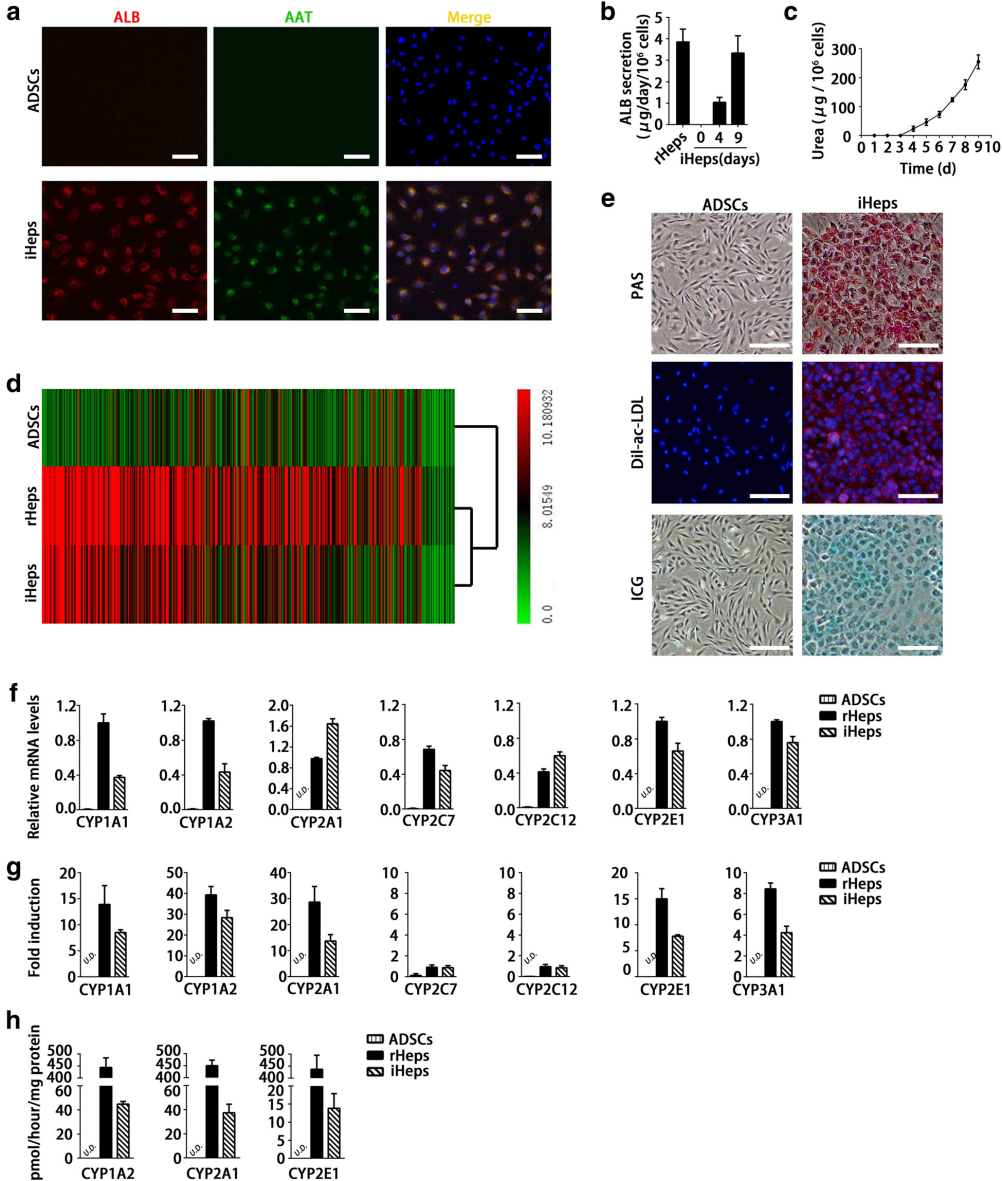
Characterization of iHeps in vitro. **a** Immunofluorescence analysis of ALB and AAT in iHeps. More than 90 % of iHeps efficiently expressed both ALB and AAT at day 9. **b** Secretion of ALB increased during the hepatogenic induction period as measured by ELISA. **c** The cumulative urea amounts gradually increased in the iHeps culture system during the hepatogenic induction period. **d** Gene expression profile analysis of ADSCs, iHeps, and rHeps cells by cDNA microarray. Hierarchical clustering showed that iHeps were grouped together with rHeps. **e** Analysis of basic hepatic function in iHeps, including PAS staining, ac-LDL, and ICG uptake. **f** mRNA levels of CYP genes were determined by quantitative PCR in iHeps and rHeps before inducer treatment. Data normalized to the level of GAPDH. **g** mRNA levels of the induced CYP enzymes were measured by quantitative PCR. CYP1A1and CYP1A2 was induced by 3-methylcholanthrene. CYP2A1 and CYP3A1 were induced by phenobarbital. CYP2E1 was induced by acetone. Fold induction in iHeps and rHeps was normalized to levels in cells without inducer treatment, respectively. **h** CYP metabolic activity in iHeps. CYP enzymes were induced in iHeps for 48 hours. Fresh rHeps were directly used as a positive control. The metabolic products of phenacetin (acetaminophen, assay for CYP1A2 activities), coumarin (7-hydroxycoumarin, assay for CYP2A1 activities), and chlorzoxazone (6-hydroxychlorzoxazone, assay for CYP2E1 activities) were determined by liquid chromatography–tandem mass spectrometry. Scale bar: 100 μm (**a**, **e**). *AAT* alpha-1-antitrypsin, *ac-LDL* acetylated low-density lipoprotein, *ADSC* adipose-derived stem cell, *ALB* albumin, *CYP* cytochrome P, *ICG* indocyanine green, *iHep* induced functional hepatocyte, *PAS* Periodic acid–Schiff, *rHep* primary rat hepatocyte, *UD* undetectable

Immunofluorescent staining showed that more than 90 % of iHeps expressed both ALB and AAT at day 9 (Fig. 3a), suggesting a highly-efficient hepatogenic differentiation. To assess the metabolic activity of iHeps, we quantified ALB production and urea synthesis. We found that iHeps had a remarkable capability for secreting the plasma protein ALB at a level close to rHeps at day 9 (Fig. 3b). The cumulative urea amounts also gradually increased in the iHep culture system during the induction period (Fig. 3c). Genome-wide expression profile analysis revealed that iHeps were clustered closely with cultured rHeps (Fig. 3d). Furthermore, gene set enrichment analysis showed that pathways enriched in rHeps were significantly enriched in iHeps, including those involved in glucose metabolism, lipid metabolism, amino acid metabolism, and phase I and phase II detoxification (see Additional file 2). These data indicate that ADSCs undergo hepatic differentiation by transcriptional alterations. In accordance with the expression of hepatic genes, iHeps displayed numerous hallmark functions of mature hepatocytes, such as glycogen storage, ac-LDL intake, and ICG uptake (Fig. 3e). The drug metabolic capacity is one of the most important functions that distinguish hepatocytes. CYP450 enzymes of hepatocytes are the main enzymes accounting for drug metabolism. Their activities and responses to specific inducers are used to evaluate drug metabolism of hepatocytes. We quantitatively confirmed the expression of CYP enzymes in iHeps, CYP1A1, CYP1A2, CYP2A1, CYP2C7, CYP2C12, CYP2E1, and CYP3A1. Results showed that iHeps already expressed these genes at remarkable levels without addition of chemical inducers (Fig. 3f). Furthermore, chemical inducers (3-methylcholanthrene, phenobarbital, and acetone) could markedly induce mRNA expression levels of CYP1A1, CYP1A2, CYP2A1, CYP2E1, and CYP3A1, except for CYP2C7 and CYP2C12 (Fig. 3g). Importantly, in another assay for CYP activities, iHeps displayed CYP enzyme-dependent metabolism of phenacetin, coumarin, and chlorzoxazone (Fig. 3h). These results offer the possibility of using iHeps for toxicity screening during drug discovery.

### iHeps improve CCl_4_-induced acute fulminant liver failure

**Fig. 4:**
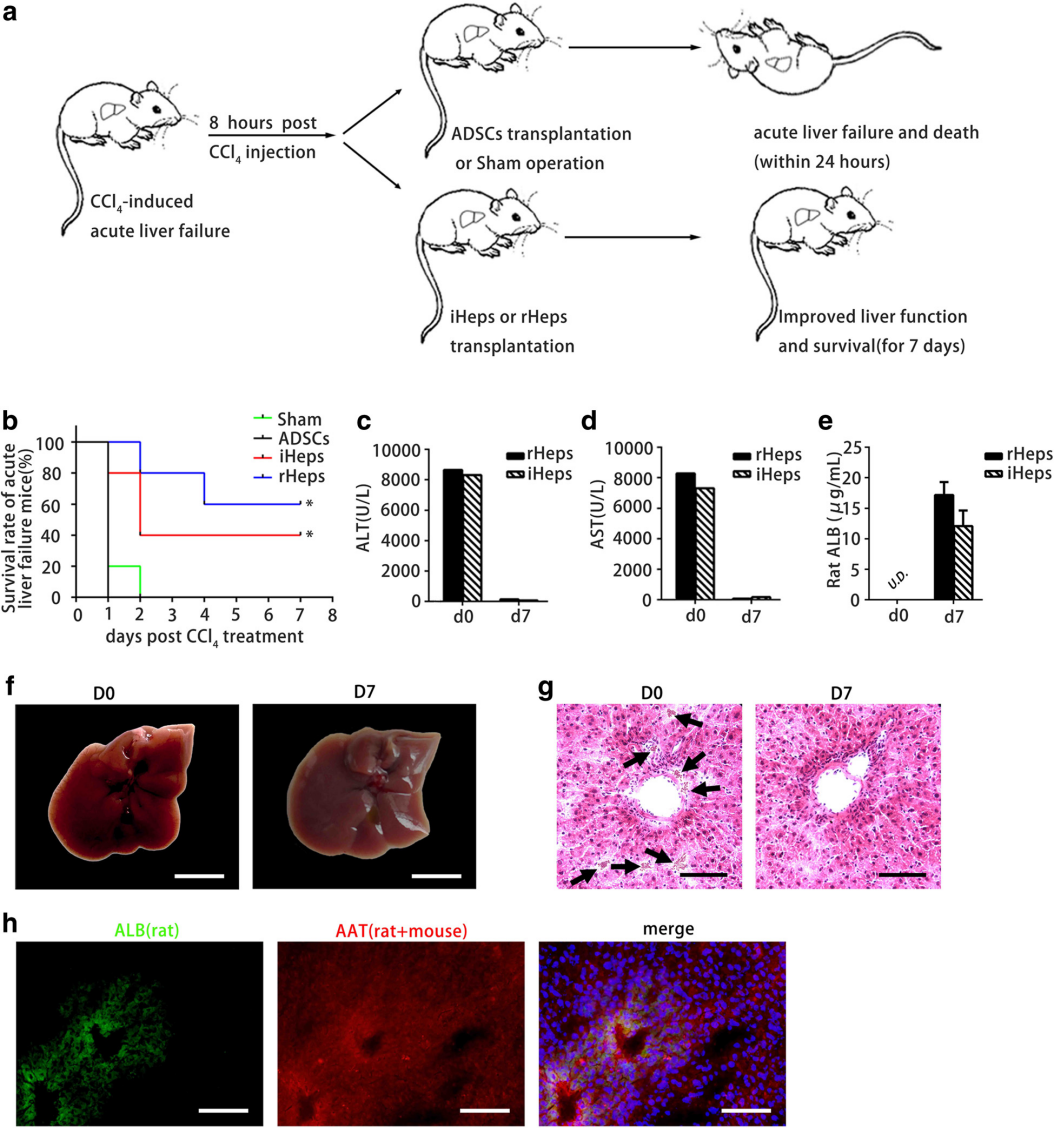
Therapeutic effects of iHeps on acute liver failure. **a** Schematic diagram of iHep transplantation into the livers of NPG mice. NPG mice were injected with carbon tetrachloride (*CCl*_*4*_) to trigger fulminant hepatitis, which led to acute liver failure and death within 24 hours in all mice. Eight hours after CCl_4_ treatment, ADSCs, iHeps, and rHeps (2 × 10^6^ cells/animal) were injected into the spleens of the mice. **b** Kaplan–Meier survival curve of NPG mice with acute liver failure that did not receive cells (sham operation) or received 2 × 10^6^ ADSCs, iHeps, and rHeps. Almost all mice died in groups transplanted with ADSCs or sham operation (one mouse in the sham operation group died at day 2), two of five recipient mice survived after transplantation with iHeps, and three of five mice receiving rHep transplantation survived. Kaplan–Meier survival curve depicted. **c**, **d** Serum levels of ALT (**c**) and AST (**d**) in CCL_4_-treated mice before (day 0) and after (day 7) transplantation of iHeps or rHeps. **e** Rat ALB levels were determined by ELISA in the sera of surviving NPG mice. **f**, **g** Livers in CCl_4_-treated mice before (day 0) and after iHep transplantation (day 7). Macroscopic images of freshly isolated livers (**f**) and hematoxylin and eosin staining of liver sections (**g**). Note CCl_4_-induced hepatitis and hemorrhage in the liver at day 0 (**f**), *arrows* in (**g**). The liver already recovered from CCl_4_-induced damage at day 7. **h** Expression of ALB and AAT in engrafted iHeps revealed by immunofluorescence. The ALB antibody is rat specific and the AAT antibody reacts with both rat and mouse. Data presented as mean ± standard deviation. Scale bar: 5 mm (**f**), 100 μm (**g**, **h**). *AAT* alpha-1-antitrypsin, *ADSC* adipose-derived stem cell, *ALB* albumin, *ALT* alanine transaminase, *AST* glutamic-oxaloacetic transaminase, *iHep* induced functional hepatocyte, *PAS* Periodic acid–Schiff, *rHep* primary rat hepatocyte

To evaluate whether iHeps have sufficient hepatic functions to support the liver in recovering from acute fulminant hepatitis, NPG mice (T, B, and natural killer cell-depleted mice) were injected with CCl_4_ to trigger acute fulminate hepatitis and animal death within 24 hours (Fig. 4a). ADSCs, iHeps, and rHeps were separately injected intrasplenically into NPG mice 8 hours after CCl_4_ treatment (Fig. 4a). In groups of mice transplanted with ADSCs or sham operation, almost all recipients died within 24 hours after CCl_4_ treatment (one mouse in the sham operation group died at day 2; Fig. 4b). rHep transplant significantly improved the survival rate and extended the survival time of mice with acute liver failure (Fig. 4b). Remarkably, upon transplant with iHeps, two of five mice completely recovered from CCl_4_-induced acute fulminant liver failure (Fig. 4b) and showed normal serum alanine transaminase (ALT) and glutamic-oxaloacetic transaminase (AST) levels on day 7 after CCl_4_ treatment (Fig. 4c, d). Rat ALB was also detected in the sera of mice transplanted with iHep cells (Fig. 4e). Histological analysis revealed that iHeps significantly improved recovery from CCl_4_-induced liver damage (Fig. 4f, g). Immunofluorescence staining of rat ALB showed the repopulation of iHeps in the liver parenchyma in the surviving mice, and repopulated ALB-positive cells were positively stained by AAT, an antibody labeling both rat and mouse hepatocytes (Fig. 4h). Remarkably, iHeps did not fuse with mouse hepatocytes as determined by immunofluorescent staining using antibodies specifically against rat ALB and mouse HNF4α (see Additional file 3). These results indicate that iHeps can populate the liver and have a therapeutic effect in the treatment of acute fulminant liver failure. Intriguingly, repopulated iHeps were not proliferating as shown by Ki67 staining 8 weeks after transplantation (see Additional file 3). Furthermore, we found that iHeps did not form tumors after transplantation in immunodeficient mice (see Additional file 4). Together, our data indicate that iHeps possess notable mature hepatocyte functions to support the liver in recovering from acute fulminant failure.

Several studies have described the differentiation of ADSCs into cells that display hepatocytic characteristics [27–30]. However, those in vitro differentiation methods are not applicable to practical use because about 1 month is required to induce ADSCs into cells with hepatic functions. Applications in the current study require a special approach, such as shortening as much as possible, including cultivation and direct hepatic fate. In this work, we presented a protocol which allowed ADSCs to differentiate into functional hepatocytes in a short time. Nine days is sufficient to obtain cells, which shows hepatocyte-specific morphology, expression profiles, and functions. To our knowledge, this is the first time such a short hepatogenic differentiation protocol has been presented. In this protocol, ADSCs are first exposed to a high level of transforming growth factor beta (TGF-β)/activin/nodal (IDE1) and Wnt (CHIR99021) signaling in a manner that is designed to mimic events during embryonic development in order to allow DE formation [31]. IDE1 is a small molecule compound and could induce DE differentiation up to 80 % of mouse embryonic stem cells (mESCs) (or 50 % of human embryonic stem cells (hESCs)) in the absence of activin A (a typically used DE inducer) [32, 33]. Similar to activin A, IDE1 induces Smad2 phosphorylation in mESCs, while their targets remain unknown. In our study, a combination of IDE1 and CHIR99021 (a specific chemical inhibitor of GSK3) was able to efficiently drive ADSCs in a definitive commitment to endoderm formation. Compared with cells cultured in media without CHIR99021 (data not shown), we found that the presence of CHIR99021 might have a synergistic effect with IDE1 and was able to efficiently induce a rapid increase in the expression of the DE markers Sox17 and FoxA2. The cell morphology of ADSCs also quickly changes into a short spindle shape within 1 day. This enables ADSCs to differentiate into hepatocytes quickly and efficiently. Unlike the recent studies [34–36], our data showed that more than 90 % of iHeps efficiently expressed both ALB and AAT and secreted the plasma protein ALB at a level close to primary rHeps after 9 days of induction. Furthermore, iHeps possessed remarkable CYP enzyme activity which was associated with drug metabolism. Additionally, transplanted iHeps could repopulate livers of NPG mice with acute fulminant liver failure and restore the liver function. Taken together, our study provides a simple, rapid, and high-efficiency protocol for the generation of ADSC-derived iHeps suitable for cell-based therapy, as well as an in vitro drug screening model.

## Conclusion

In summary, our study outlines rapid generation of mature functional hepatocyte cells from ADSCs by an efficient three-step induction protocol. Furthermore, we have shown therapeutic effects of these cells on CCl_4_-induced acute fulminant liver failure. This work could contribute to the development of alternative strategies to obtain nonhepatic cell-derived mature hepatocytes with potential for biomedical and pharmaceutical applications.

## Additional files

Additional file 1: Table S1. Showing forward (F) and reverse (R) primer pairs used for real-time quantitative PCR, related to Figs. 2 and 3. (PDF 138 kb) Additional file 2: Table S2. Showing hepatic pathways enriched in rHeps and iHeps cells, related to Fig. 3. (PDF 98 kb) Additional file 3: Figure S1. Showing transplantation of iHeps into NPG mice, related to Fig. 4. A Cell fusion between repopulated iHeps and recipient mouse hepatocytes were excluded by co-staining of rat AlB (*green*) and mouse HNF4α (*red*). Antibody specific for rat ALB stained iHeps, while antibody specific for mouse HNF4α stained mouse hepatocytes. B Sections of NPG livers 8 weeks after iHep transplantation were stained by rat ALB (*green*), rat and mouse Ki67 (*red*) antibodies. iHeps were positively stained for rat ALB, but were negatively stained for rat and mouse Ki67. Scale bars: 50 μm A, B. (PDF 193 kb) Additional file 4: Figure S2. Showing transplantation of iHeps into BALB/c nude mice, related to Fig. 4. HepG2 (1 × 10^6^) or iHeps (1 × 10^6^) cells were subcutaneously transplanted into the bank areas of nude mice. iHeps cells did not form tumors 8 weeks after transplantation. (PDF 118 kb)

## Abbreviations

AAT: Alpha-1-antitrypsin
ac-LDL: Acetylated low-density lipoprotein
ADSC: Adipose-derived stem cell
ALB: Albumin
ALT: Alanine transaminase
ASGPR: Asialoglycoprotein receptor
AST: Glutamic-oxaloacetic transaminase
BSA: Bovine serum albumin
CCl_4_: Carbon tetrachloride
CYP: Cytochrome P
DAPI: 4',6-Diamidino-2-phenylindole
DE: Definitive endoderm
DEX: Dexamethasone
DMEM-LG: Low-glucose Dulbecco’s modified Eagle’s medium
EGF: Epidermal growth factor
ELISA: Enzyme-linked immunosorbent assay
FBS: Fetal bovine serum
FGF4: Fibroblast growth factor 4
HBSS: Hank’s balanced salt solution
HGF: Hepatocyte growth factor
HNF4α: Hepatocyte nuclear factor alpha-4
ICG: Indocyanine green
iHEp: Induced functional hepatocyte
ITS: Insulin–transferrin–selenium
KEGG: Kyoto Encyclopedia of Genes and Genomes
MSC: Mesenchymal stem cell
OsM: Oncostatin M
PAS: Periodic acid–Schiff
PBS: Phosphate-buffered saline
rHep: Primary rat hepatocyte
TGF-β: Transforming growth factor beta
