# Supplementary material for: Rapid and high-efficiency generation of mature functional hepatocyte-like cells from adipose-derived stem cells by a three-step protocol
Source: Stem Cell Res Ther. 2015 Oct 5;6:193. doi: 10.1186/s13287-015-0181-3 (PMC4595267; doi:10.1186/s13287-015-0181-3)
Supplement: Additional file 1: Table S1. — Showing forward (F) and reverse (R) primer pairs used for real-time quantitative PCR, related to Figs. 2 and 3. (PDF 138 kb) [file 13287_2015_181_MOESM1_ESM.pdf]

Table S1 Forward (F) and reverse (R) primer pairs used for real-time quantitative PCR, related to Figure 2 and Figure 3.

Genes (ID)	Sequence (5'-3')
GAPDH(NM_017008.4)	F: ATGGTGAAGGTCGGTGTGAA
	R: CACTTTGTCACAAGAGAAGGCAG
AAT(NM_022519.2)	F: CATCACCCGGGTCTTCAACA
	R: CTTCACTTGAGGGGGCAGAG
ALB(NM_134326.2)	F: ACACCCAGAAAGCACCTCAG
	R: CACACACAGACGGTTCAGGA
ASGPR1(NM_012503.2)	F: CTGCGGGTTCTAAGGCAGAA
	R:TTCCCACTCTCTCTCCCTGG
GJB1(NM_017251.2)	F: ATCTTGGTTTCCACCCCAGC
	R: CCGGAACACCACACTGATGA
CK18(NM_053976.1)	F: GATATCCGTGTCCCGCTCTG
	R: CACCTTGTCTAGGTAGCTGGC
TTR(NM_012681.2)	F: GAAGTTCACGGAAGGGGTGT
	R: CGATGGTGTAGTGGCGATGA
Transferrin(NM_001013110.1)	F: TTCCAACTGTTCGGCTCTCC
	R: GCCTGTAGTCCATCCTTGGG
TAT(NM_012668.2)	F: AAGTCCAATGCGGACCTCTG
	R: TCAACCGCTCTGTGAACTCC
HNF4α(NM_001270933.1)	F: GTGGCGAGTCCTTATGACACAT
	R: GAGGCTCCGTAGTGTTTGCC
AFP(NM_012493.2)	F: CCAGTGCCCGACAGAGAAAA
	R: TACTTCGGCCTTTGTGGCAT
CYP1A1(NM_012540.2)	F: AGACACAGTGATTGGCAGGG
	R: TGTCGGAAGGTCTCCAGGAT
CYP3A1(NM_013105.2)	F: TCTGTTTGCCATCACGGACA
	R: AATCCCCACTGGGCCAAAAT
CYP2A1(NM_012692.1)	F: GAGGCGAACAGGCTACCTAC
	R: CTGAGTTGTTTTGCCCGCTC
CYP2E1(NM_031543.1)	F: TTCACCAAGTTGGCAAAGCG
	R: CCTTGACAGCCTTGTAGCCA
CYP2C7(NM_017158.2)	F: TCTCTGGTTCACCCAAAGGAC
	R: AAGCAGAGCTGGTAAGTGGG
CYP2C12(NM_031572.1)	F: ACGGAAATGTGTGGGAGAGG
	R: TCGATGTCCTTTGGATCAGACA
CYP1A2(NM_012541.3)	F: GAGCGAGGAGATGCTCAACC
	R: GAAGTCCACAGCATTCCCTGA
