# Supplementary material for: Rapid and high-efficiency generation of mature functional hepatocyte-like cells from adipose-derived stem cells by a three-step protocol
Source: Stem Cell Res Ther. 2015 Oct 5;6:193. doi: 10.1186/s13287-015-0181-3 (PMC4595267; doi:10.1186/s13287-015-0181-3)
Supplement: Additional file 2: Table S2. — Showing hepatic pathways enriched in rHeps and iHeps cells, related to Fig. 3. (PDF 98 kb) [file 13287_2015_181_MOESM2_ESM.pdf]

Table S2 Hepatic pathways enriched in rHeps and iHeps cells, related to Figure 3.

Enriched pathways	rHeps		iHeps	
	FDR q-Val	NES	FDR q-Val	NES
Adipocytokine signaling pathway	0.0499851	2.087687	0.05410332	2.318348
Arginine and proline metabolism	0.001199262	4.188258	0.1385875	1.618363
Bile secretion	0.01472299	2.83033	0.06067794	2.198905
Biosynthesis of unsaturated fatty acids	0.001199262	4.210528	0.03064826	3.081796
Drug metabolism - cytochrome P450	0.00000	6.549075	0.04944426	2.656177
Fatty acid elongation	0.0499851	2.089091	0.05410332	2.545572
Fatty acid metabolism	0.00000	7.716632	0.1121858	1.750578
Glutathione metabolism	0.00000	5.779929	0.001500476	5.237071
Glycerophospholipid metabolism	0.0288329	2.448261	0.1947902	1.360305
Glycosaminoglycan biosynthesis - chondroitin sulfate / dermatan sulfate	0.1112854	1.567521	0.2107233	1.304257
Glycosaminoglycan biosynthesis - keratan sulfate	0.1219354	1.514257	0.1003299	1.828563
Peroxisome	0.00000	7.269767	0.06362403	2.132557
PPAR signaling pathway	0.00000	6.63638	0.04944426	2.640003
Primary bile acid biosynthesis	0.001710999	3.97593	0.1219661	1.703811
Steroid biosynthesis	0.05909453	1.951556	0.06425409	2.114037
Synthesis and degradation of ketone bodies	0.04186058	2.20043	0.1003299	1.835649
Sulfur metabolism	0.06346566	1.878367	0.01875716	3.441163
Metabolism of xenobiotics by cytochrome P450	0.00000	7.766024	0.05410332	2.334036
