# Supplementary material for: Rapid and high-efficiency generation of mature functional hepatocyte-like cells from adipose-derived stem cells by a three-step protocol
Source: Stem Cell Res Ther. 2015 Oct 5;6:193. doi: 10.1186/s13287-015-0181-3 (PMC4595267; doi:10.1186/s13287-015-0181-3)
Supplement: Additional file 3: Figure S1. — Showing transplantation of iHeps into NPG mice, related to Fig. 4. A Cell fusion between repopulated iHeps and recipient mouse hepatocytes were excluded by co-staining of rat AlB (green) and mouse HNF4α (red). Antibody specific for rat ALB stained iHeps, while antibody specific for mouse HNF4α stained mouse hepatocytes. B Sections of NPG livers 8 weeks after iHep transplantation were stained by rat ALB (green), rat and mouse Ki67 (red) antibodies. iHeps were positively stained for rat ALB, but were negatively stained for rat and mouse Ki67. Scale bars: 50 μm A, B. (PDF 193 kb) [file 13287_2015_181_MOESM3_ESM.pdf]

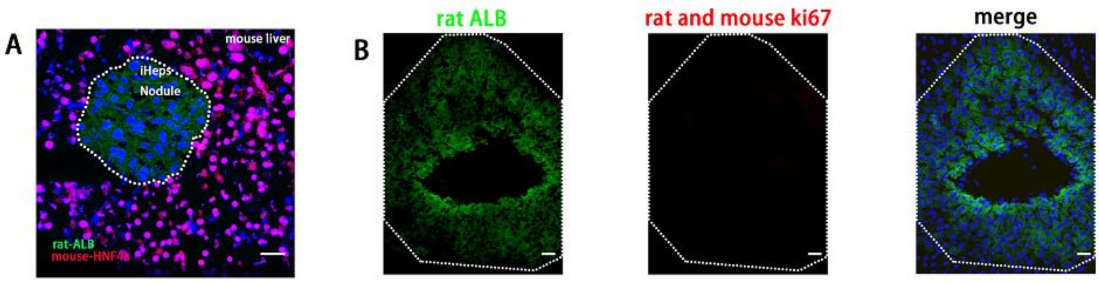

Figure S1 Transplantation of iHeps into NPG mice, related to Figure 4. (A) Cell fusion between repopulated iHeps and recipient mouse hepatocytes were excluded by co-staining of rat AlB (Green) and mouse HNF4α (Red). Antibody specific for rat ALB stained iHeps, while antibody specific for mouse HNF4α stained mouse hepatocytes. (B) Sections of NPG livers 8 weeks after iHeps transplantation were stained by rat ALB (Green), rat and mouse Ki67 (Red) antibodies. iHeps were positively stained for rat ALB, but were negatively stained for rat and mouse Ki67. Scale bars: 50 μm (A, B).
