# Supplementary material for: Rapid and high-efficiency generation of mature functional hepatocyte-like cells from adipose-derived stem cells by a three-step protocol
Source: Stem Cell Res Ther. 2015 Oct 5;6:193. doi: 10.1186/s13287-015-0181-3 (PMC4595267; doi:10.1186/s13287-015-0181-3)
Supplement: Additional file 4: Figure S2. — Showing transplantation of iHeps into BALB/c nude mice, related to Fig. 4. HepG2 (1 × 10^6^) or iHeps (1 × 10^6^) cells were subcutaneously transplanted into the bank areas of nude mice. iHeps cells did not form tumors 8 weeks after transplantation. (PDF 118 kb) [file 13287_2015_181_MOESM4_ESM.pdf]

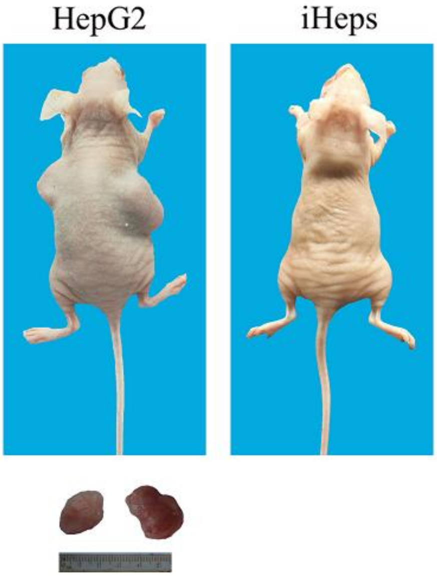

	Tumor-bearing mice per injected mice
HepG2	10/5
iHeps	0/5

Figure S2 Transplantation of iHeps into BALB/c nude mice, related to Figure4. HepG2 (1×10^6) or iHeps (1×10^6) cells were subcutaneously transplanted into the bank areas of nude mice. iHeps cells did not form tumors 8 weeks after transplantation.
